# Molecular sexing of threatened Gyps vultures: an important strategy for conservation breeding and ecological studies

**DOI:** 10.1186/2193-1801-1-62

**Published:** 2012-12-12

**Authors:** Prabhakar B Ghorpade, Praveen K Gupta, Vibhu Prakash, Richard J Cuthbert, Mandar Kulkarni, Nikita Prakash, Asit Das, Anil K Sharma, Mohini Saini

**Affiliations:** 1 Centre for Wildlife Conservation, Management & Disease Surveillance, Indian Veterinary Research Institute, Izatnagar, 243 122 India; 2 Division of Veterinary Biotechnology, Indian Veterinary Research Institute, Izatnagar, 243 122 India; 3 Bombay Natural History Society, Hornbill House, S.B. Singh Road, Mumbai, 400 001 India; 4 Royal Society for the Protection of Birds, The Lodge, Sandy, Bedfordshire, UK

**Keywords:** Molecular sex identification, *Gyps* vulture, Cinereous vulture, Vulture conservation, Captive breeding

## Abstract

During the last two decades populations of three resident species of *Gyps* vulture have declined dramatically and are now threatened with extinction in South Asia. Sex identification of vultures is of key importance for the purpose of conservation breeding as it is desirable to have an equal sex ratio in these monogamous species which are housed together in large colony aviaries. Because vultures are monomorphic, with no differences in external morphology or plumage colour between the sexes, other methods are required for sex identification. Molecular methods for sex identification in birds rely on allelic length or nucleotide sequence discrimination of the chromohelicase-DNA binding (*CHD*) gene located on male and female chromosomes *ZZ* and *ZW*, respectively. We characterized the partial sequences of *CHD* alleles from *Gyps indicus*, *Gyps bengalensis*, *Gyps himalayensis* and *Aegypius monachus* and analysed the applicability of five molecular methods of sex identification of 46 individual vultures including 26 known-sex *G. bengalensis* and *G. indicus*. The results revealed that *W*-specific PCR in combination with *ZW*-common PCR is a quick, accurate and simple method, and is ideal for sex identification of vultures. The method is also suitable to augment ecological studies for identifying sex of these endangered birds during necropsy examinations especially when gonads are not apparent, possibly due to regression during non-breeding seasons.

## Background

Nine species of vultures in the family Accipitridae are found in India, three of which are endemic to South and South-East Asia (the Oriental white-backed vulture (*Gyps bengalensis*), long-billed (*G. indicus*) and slender-billed vulture (*G. tenuirostris*) and are classified as Critically Endangered by the International Union for Conservation of Nature and Natural resources and are at high risk of extinction in the wild (IUCN, 2011). In India, populations of *G. bengalensis* have declined by more than 99.9% while those of *G. indicus* and *G. tenuirostris* have declined by around 97% between the early 1990s and 2007 (Prakash et al. 2007). Similar reductions in vulture populations have been recorded in Pakistan and Nepal (Pain@ et al. 2008). Although the Himalayan griffon (*G. himalayensis*) is not considered threatened (under category Least Concern) (IUCN, 2011), its population decline has been recorded in Nepal (Acharya et al. 2009). The status of another species, the Cinereous Vulture (*Aegypius monachus*), is classified as Near Threatened as per IUCN (IUCN, 2011). Veterinary use of non-steroidal anti-inflammatory drugs (NSAIDs) such as diclofenac and ketoprofen have been shown to be toxic to *Gyps* vultures and are responsible for the decline of these species (Oaks et al. 2004; Green et al. 2006, 2007; Swan et al. 2006; Cuthbert et al. 2009; Naidoo et al. 2009, 2010; Das et al. 2011). In contrast the NSAID meloxicam has been demonstrated to be a safe and effective alternative drug for veterinary use (Swan et al. 2006; Swarup et al. 2007). Although the veterinary use of diclofenac has been banned in India, Pakistan and Nepal (Kumar 2006; Singh 2008), it’s illegal use is still apparent, as diclofenac residues are still prevalent in cattle carcasses across India at concentrations sufficient to cause declines in vulture populations (Cuthbert et al. 2011a, 2011b; Saini et al. 2012).

Due to the massive scale of the population declines and the continued use of diclofenac, populations of the three Critically Endangered resident *Gyps* species are being bred in captivity in India, Nepal and Pakistan, with the aim that their progeny will be introduced back in to the wild after ensuring that the environment is safe and diclofenac free (MoEF 2006; Bowden 2009).

Vultures are monomorphic monogamous species and hence without knowing the sex of birds it is difficult to maintain the correct sex ratios in aviaries at conservation breeding centres in order to maximise the chances of successful breeding. As well as the key importance of identifying gender for conservation breeding programmes, knowledge of sex is also important to complement forensic studies (An et al. 2007) and investigations on evolution and ecology (Griffiths and Tiwari 1995; Costantini 2008; Fukui et al. 2008).

Various techniques have been employed for sex determination of monomorphic birds such as laparotomy (Risser 1971), laparoscopy (Richner 1989), flow cytometry (Nakamura et al. 1990), karyotyping (Hatzofe and Getreide 1990) and Raman spectroscopy (Harz et al. 2008) but molecular methods based on DNA analysis are most prevalent (Fridolfsson and Ellegren 1999). Except for the ratites, that have undifferentiated sex chromosomes, all male birds are homogametic with *ZZ* sex chromosomes and females are heterogametic with *ZW* sex chromosomes (Ellegren 1996; Griffiths et al. 1996). The most frequently exploited gene for sex identification is the Chromohelicase DNA binding (*CHD*) gene that is found conserved on both W and Z chromosomes (Griffiths 2000). Intronic length variation in *CHD-Z* and *CHD-W* allelles amplified by Griffiths universal *CHD* primer pair P2/P8 has formed the basis of gender identification in most avian species (Griffiths et al. 1998; Fridolfsson and Ellegren 1999). However, in certain species of Accipitridae there is an extremely short difference in intronic length between *CHD-Z* and *CHD-W* P2/P8 amplicon which makes sex identification more difficult and inaccurate (Ito et al. 2003; Chang et al. 2008). Hence, in order to circumvent the limitation of conventional PCR (Fridolfsson and Ellegren 1999), different approaches detecting small variation in nucleotides like Amplification Refractory Mutation System (ARMS) (Ito et al. 2003), Restriction Fragment Length Polymorphism (RFLP) (Sacchi et al. 2004), Single strand conformation polymorphism (SSCP) (Ramos et al. 2009), Melting Curve analysis (Chang et al. 2008a), *ZW* common and *W*-specific PCR (Chang et al. 2008b), TaqMan Probe-based real time PCR (Chang et al. 2008c; Chou et al. 2010) have been suggested in order to identify gender in these species.

Old World vultures along with other birds of prey belong to the taxonomic order Falconiformes, family Accipitridae, and subfamily Accipitrinae (Chang et al. 2008b, 2008c). Due to their position within the Accipitridae it was observed that intronic length variation of *CHD-Z* and *CHD-W* amplicon in Griffiths universal *CHD* primer pair P2/P8 based PCR is unlikely to be suitable for sex discrimination in *G. indicus* or *G. bengalensis* (Reddy et al. 2007). However, a similar approach (Kahn et al. 1998) using denaturing polyacrylamide gel electrophoresis combined with autoradiography has been reported to sex nestlings of these two species (Arshad et al. 2009). In the present study, based upon the chromohelicase gene sequences in male (*ZZ*) and female birds (*ZW*), the accuracy and reliability of five different approaches are compared for molecular gender identification in three vulture species (*G. indicus, G. bengalensis, G. himalayensis*) in order to identify an accurate and simple test to support the captive breeding programmes.

## Results

### Sequence characterization of *CHD-Z* and *CHD-W* sequences

**Figure 1:**
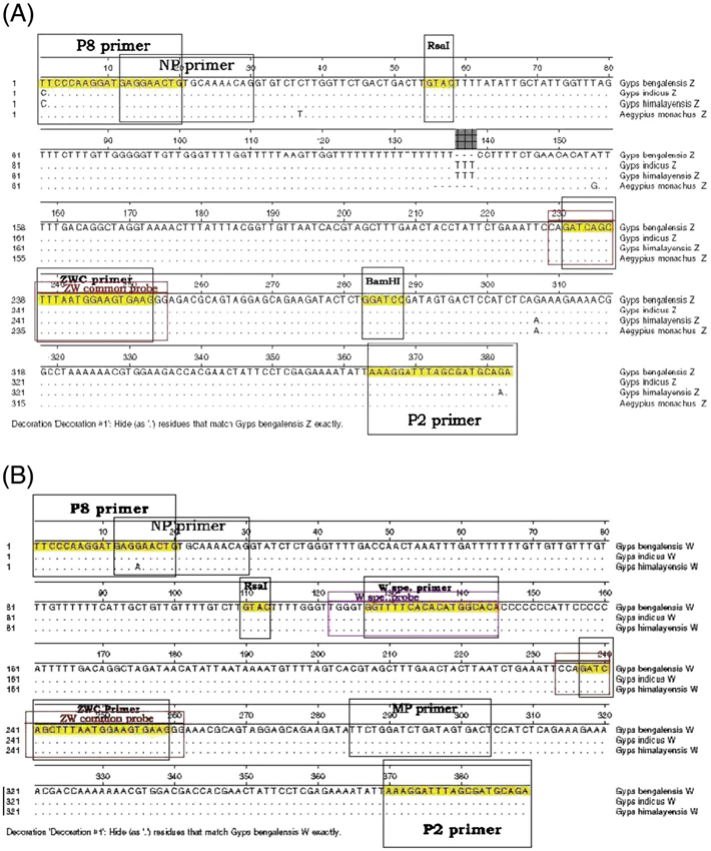
**Sequence alignment of*CHD-Z*and*CHD-W*allele sequences amplified by Griffith’s universal*CHD*primer pair P2/P8 in four species of vultures (*Gyps bengalensis*,*Gyps indicus*,*Gyps himalayensis*,*Aegypius monachus*) by ClustalW (MegAlign DNAstar).** The sequences of primers P2, P8, NP, MP, *ZW* common primer and probe, *W*-specific primer and probe recognition sites; *Bam*HI and *Rsa*I restriction sites are boxed. (−−) Dashed lines indicate sequence similarity. **A**.) Alignment of *CHD-Z* sequences. **B**.) Alignment of *CHD-W* sequences.

The *CHD-Z* and *CHD-W* sequences from the four vulture species used in this study were amplified and the sequences were determined. These sequences were submitted to GenBank and accession numbers obtained were HQ236387, HQ236386 (*G. indicus*); HQ236388, HQ236385 (*G. bengalensis*); HQ236384, HQ236383 (*G. himalayensis*); HQ236382 (*A. monachus*). Independent alignment reports for *CHD-Z* and *CHD-W* sequences were prepared (Figure 1A and B), where primer binding regions for P2, P8, NP, MP; *ZW*-Common and *W*-specific primers and probes as well as restriction site for *Bam*HI and *Rsa*I were located. The primer binding region for MP and *W*-specific primers were found in all *CHD-W* but not in *CHD-Z* sequences. The recognition sequence for *Bam*HI was found on *CHD-Z* but was absent on the *CHD-W* sequence, whereas the *Rsa*I restriction site was located at different positions in the *CHD-Z* and *CHD-W* sequences. Based on these identified sequences the applicability and accuracy of PCR-RFLP, ARMS-PCR, *W*-specific PCR and TaqMan probe based real-time PCR methods for sex identification was tested for all four species of vultures (Table 1).

**Table 1:** **Predicted gel pattern for analysing various sex identification methods (size in bp)**

P2/P8		PCR-RFLP				ARMS-PCR (Multiplex with P2/NP/MP primers)		***W***-specific PCR			
		***Bam***HI digest of P2/P8 amplicon		***Rsa***I digest of P2/P8 amplicon				P2/***ZW*** common		P2/***W*** -specific	
***Gyps bengalensis***											
Female	Male	Female	Male	Female	Male	Female	Male	Female	Male	Female	Male
389(*W*)	Nil	389		327	327	378(P2/NP-*W*)		153(*W*)		263(*W*)	Nil
383(*Z*)	383(*Z*)	283	283	278		372(P2/NP-*Z*)	372(P2/NP-*Z*)	153(*Z*)	153(*Z*)		
		100	100	111		293(MP/NP-*W*)	Nil				
				56	56						
***Gyps indicus***											
389(*W*)	Nil	389		330	330	378(P2/NP-*W*)		153(*W*)		263*(W*)	Nil
386(*Z*)	386(*Z*)	286	286	278		375(P2/NP-*Z*)	375(P2/NP-*Z*)	153(*Z*)	153(*Z*)		
		100	100	111		293(MP/NP-*W*)	Nil				
				56	56						
***Gyps himalayensis***											
389(*W*)	Nil 386(*Z*)	389		330	330	378(P2/NP-*W*)		153(*W*)		263(*W*)	Nil
386(*Z*)		286	286	278		375(P2/NP-*Z*)	375(P2/NP-*Z*)	153(*Z*)	153(*Z*)		
		100	100	111		293(MP/NP-*W*)	Nil				
				56	56						
***Aegypius monachus***											
-	380(*Z*)	-	280	-	324	-	369(P2/NP-*Z*)	-	153(*Z*)	-	Nil
			100		56						

### Standardization of PCR-based molecular methods for sex identification

i) Conventional PCR-RFLP

**Figure 2:**
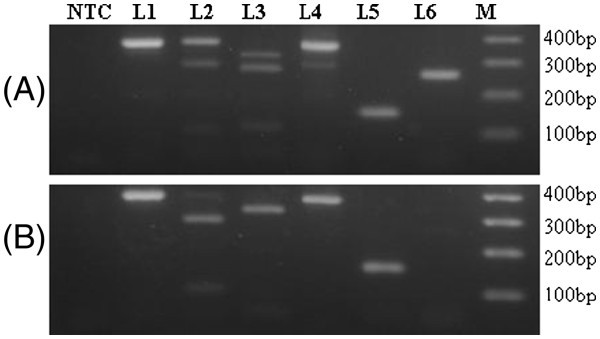
**Gel view for molecular method for sex identification of*Gyps bengalensis*using Griffith’s universal CHD primer pair P2/P8 PCR based methods:** P2/P8 PCR (L1), *Bam*HI digest of P2/P8 amplicon (L2). *Rsa*I digest of P2/P8 amplicon (L3), ARMS PCR (L4), P2/*ZW*-common PCR (L5), and P2/*W*-Specific PCR (L6). M: 100 bp DNA ladder; NTC: No template control. The PCR products and restriction digests were resolved in run 3% agarose gel electrophoresis and stained with ethidium bromide **A**. Anatomically confirmed female (P10). **B**. Anatomically confirmed male (P33).

For standardization of conventional PCR-RFLP, known sex samples from *G. bengalensis* and *G. indicus*, *G. himalayensis* and *A. monachus* were used. It was evident from sequence analysis (Figure 1A and B) and the predicted fragment pattern (Table 1) that the test employed for sex identification of *G. bengalensis*, *G. indic*us, or *G. himalayensis* is expected to produce a similar pattern on agarose gel. Figure 2 represents the results for *G. bengalensis* male (P33) and female (P10) birds. Similar patterns of results were found with other species of vultures as predicted (data not shown). PCR amplified products (383 bp in case of *CHD-Z* and 389 bp in case of *CHD-W*) as expected for *G. bengalensis* were obtained using Griffith’s universal *CHD* primer pair which could not be resolved in agarose gel (Figure 2A, L1 and 2B, L1). On restriction digestion with *Bam*HI, female *CHD* gene yielded three fragments (389 bp, 283 bp, 100 bp) (Figure 2B, L2), while male *CHD* gene yielded two fragments (283 bp, 100 bp) (Figure 2A, L2). Using *Rsa*I, there were four fragments (327 bp, 278 bp, 111 bp, 56 bp) (Figure 2B, L3) for females, and two fragments (327 bp, 56 bp) for males (Figure 2A, L3). This indicated that PCR-RFLP using either *Bam*HI or *Rsa*I could be used for sex identification in all the species of vultures.

ii) ARMS-PCR

In ARMS-PCR using P2, MP and NP primers, the male bird yielded a single amplified product of 372 bp (Figure 2A, L4) because there was only one *CHD-Z* allele while, the female bird yielded three products (378 bp, 372 bp, 293 bp) because female had two alleles namely, *CHD-Z* and *CHD-W*. Since the products 378 bp, 372 bp were very close in size, they could not be separated on 3% agarose gel and appeared as single band (Figure 2B, L4).

iii) *W*-specific PCR

In the *W*-specific PCR method of sex identification, where Griffith’s universal *CHD* primer P2 was used as the forward primer and *CHD-ZW* common primer as the reverse primer (which anneals to both the *CHD-Z* and *CHD-W* sequence) this generated one product (153 bp) with both male and female birds (Figure 2A, L5 and 2B, L5). When Griffith’s universal *CHD* primer P2 was used as a forward primer and *W-*specific primer was used as reverse primer (which anneals to only the female specific *CHD-W* allele) this yielded one product (263 bp) with female birds (Figure 2A, L6) and no product with male birds as the *W*-specific primer does not bind with the *CHD-Z* allele (Figure 2B, L6).

iv) TaqMan probe based qualitative real-time PCR (qPCR)

**Figure 3:**
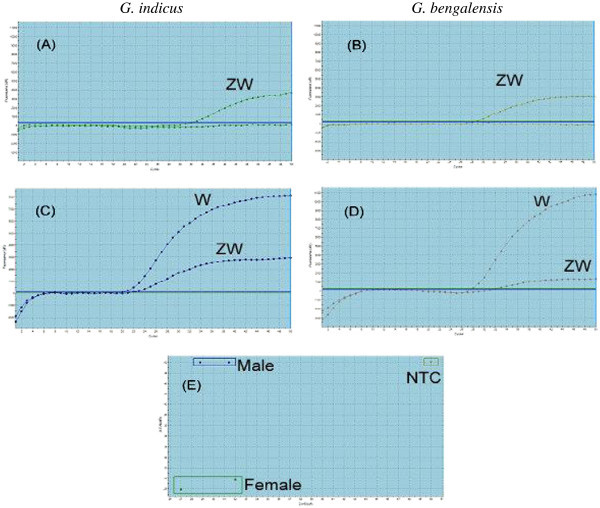
**Real-time PCR curve for sex identification of known -sex*Gyps bengalensis*(male P33, female P10) and*Gyps indicus*(male P16, female P31) using TaqMan probes.** W and ZW indicated the positive signals of TaqMan probes for *CHD-W* specific (FAM labelled) and *CHD-ZW*-common (HEX labelled) regions, respectively. ZW alone and W/ZW represented the male and female birds, respectively. **A**, **B**, **C**, **D** - Amplification plots with X-axis: PCR Cycle number, Y-axis: Fluorescence (dR). **E** - Dual color scatter plot with X- axis: Ct-HEX (dR), Y- axis Ct- FAM (dR).

Using TaqMan based qPCR based on an allele discrimination option, where Griffith’s universal *CHD* primer pair P2/P8 was used along with *ZW* common (HEX-labelled) and *W-* specific (FAM-labelled) probes, sex identification was undertaken on the basis of a colour plot. In females, where both *CHD-W* and *CHD-Z* alleles were present, both *ZW* common and *W*- specific probes gave dual colour (HEX- and FAM-specific fluorescence). While in males where only one allele (*CHD-Z*) was present, only one colour (HEX-specific fluorescence) could be detected. Results from genomic DNA of known sex *G. bengalensis* and *G. indicus* female and male birds are shown (Figure 3A-E).

### Application of the molecular methods for sex identification

**Figure 4:**
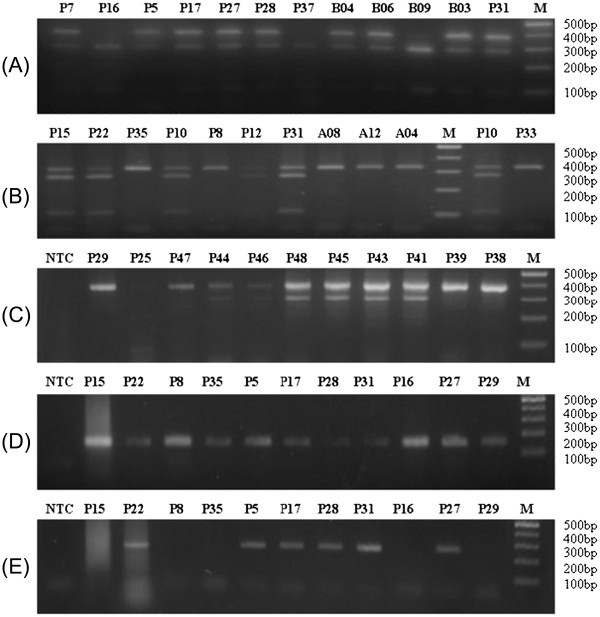
**Gel view for molecular methods for sex identification using post-mortem and live bird samples of vultures by Griffith’s universal CHD primer P2/P8 based PCR methods-** PCR- RFLP using *Bam*HI (**A**), PCR-RFLP using *Rsa*I (**B**), ARMS-PCR (**C**), P2/*ZW*-Common PCR (**D**), P2/*W*-Specific PCR (**E**). M: 100 bp DNA ladder; NTC: No template control. The PCR products and restriction digests were resolved on 3% agarose gel and stained with ethidium bromide.

36 samples (26 tissue samples and 10 blood samples) were analysed using conventional PCR with Griffith’s universal *CHD* primer pair P2/P8, a single PCR product was obtained in all samples with good quality genomic DNA. Further, using *Bam*HI and *Rsa*I restriction digestion in PCR-RFLP, sex in all samples could be successfully identified. Gel photographs of representative samples are shown (Figure 4A-B).

In ARMS-PCR (Figure 4C), where multiplexing of primers was done to analyse the sex of birds as female (with two bands) or male (with one band), sex in all samples could be identified. The female samples showed two bands of approximately 378 bp and 293 bp, whereas males yielded a single band of 372 bp in *G. bengalensis*, 375 bp in *G. indicus* as well as in *G. himalayensis*, and 369 bp in *A. monachus* as predicted in Table 1.

The *W*-specific PCR approach employing P2/*ZW* Common primer pair or P2/*W*-specific primer pair in independent reactions proved useful in identifying sex of all bird samples. P2/*ZW* common amplicon of 153 bp authenticated the *CHD* specific product obtained from all genomic DNA (Figure 4D). One band of 263 bp belonging to P2/*W*-specific product was visualized only in the female samples (Figure 4E). This method was found useful in analyzing certain samples even with degraded DNA.

**Figure 5:**
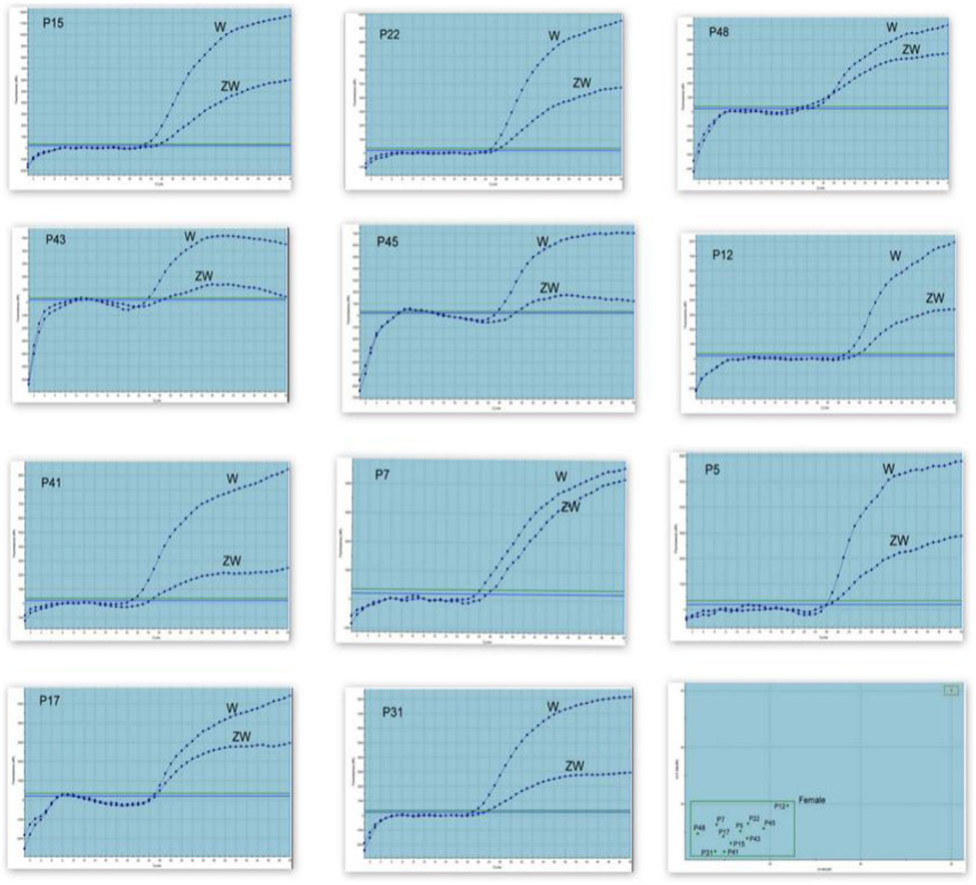
**Real-time PCR curves and scatter plot for sex identification of field necropsy specimen of*Gyps*vultures using TaqMan probes.***W* and *ZW* indicate the positive signals of TaqMan probes for CHD-*W* specific (FAM-labelled) and CHD-*ZW*-common (HEX-labelled) regions, respectively.

Similarly, the TaqMan based qPCR approach was successful in identifying sex based on allellic discrimination. Figure 5 represents the application of the Realtime qPCR to identify *Gyps bengalensis* and *Gyps indicus* female birds. The sex of the birds obtained by qPCR matched with that obtained by other agarose gel-based molecular methods.

Sex of 17 dead and nine live birds were identified by molecular methods and the results were verified from the breeding centre. Sex identified by molecular methods from 13 dead birds matched with the known sex. The sex of four samples (P7, P12, P43 and P48) that were found to be female using all of the molecular methods were previously identified as males during field post-mortems. The sexes of all the nine live birds identified by molecular methods matched with the observed sex based upon their biological behaviour in the breeding centre. The sex of a further 12 dead and eight live birds was identified based on results of the molecular methods detailed above and successfully classified sex in three *Gyps* species (*G. indicus, G. bengalensis* and *G. himalayensis*)*.*

## Discussion

To evaluate different molecular methods for sex identification of vultures we determined the *CHD-Z* and *CHD-W* gene sequences from *G. bengalensis*, *G. indicus*, *G. himalayensis* and *A. monachus* vulture species and seven sequences have been submitted to GenBank (HQ236382-HQ236388). Multiple sequence alignment of *CHD-W* and *CHD-Z* sequences revealed high sequence similarity which suggested that a common molecular method could be utilised for sex identification in all four of these vulture species (three of one genus, *Gyps* and one of different genus, *Aegyps*). Further, due to the sequence similarity of the primer binding region of Griffith’s universal *CHD* primer pair P2/P8 on the *CHD-Z* and *CHD-W* gene sequences, the amplicon from *CHD-Z* and *CHD-W* genes could be obtained in PCR. However, due to the small difference in intronic length (amplicon sizes of *CHD-Z* and *CHD-W* alleles with difference of 6 bp with *G. bengalensis* and 3 bp with *G. indicus* and *G. himalayensis*), it was not possible to differentiate males and females using standard agarose gel electrophoresis. Similar findings have been reported in other species of birds and in particular among raptors (Fridolfsson and Ellegren 1999; Ito et al. 2003; Sacchi et al. 2004; Reddy et al. 2007; Chang et al. 2008b, 2008c; Chou et al. 2010). Of the four species of vultures used in the present study, *A. monachus* is not being maintained in captivity. Only one tissue sample of this species was available that was collected from post-mortem of a single bird carcass available in the field. Considering the limitation in number of samples for *A. monachus*, we have included only the characterisation of *CHD-Z* sequence obtained from one male bird for use in application of molecular methods. The proposed methodologies are likely to be used for sex differentiation from more field specimens of cinereous vultures in future.

To discriminate *CHD-Z* and *CHD-W* amplicons and to identify male and female vultures, we analysed four different molecular approaches which have been reported as useful in differentiating sex of eagles and falcons (Ito et al. 2003; Sacchi et al. 2004; Chang et al. 2008b, 2008c; Chou et al. 2010; Reddy et al. 2007; Nesje and Roed 2000; Busch et al. 2005).

On analysis of nucleotide sequences for *CHD-Z* and *CHD-W* amplicons for the *Rsa*I restriction site, two fragments with *CHD-Z* amplicon and two fragments of different length with *CHD-W* amplicon were predicted. PCR-RFLP in our study, yielded sex differentiating fragment patterns as digestion of P2/P8 amplicon by *RsaI* produced four fragments in the case of female birds and two fragments for males. Similarly, PCR-RFLP using *Bam*HI yielded three fragments with females and two with males. Digestion of P2/P8 PCR product using *Dra*I *and Rsa*I restriction enzymes did not yield RFLP pattern expected from analysis of sequences previously published for the species *G. indicus* (DQ156155 and DQ156156) and *G. bengalensis* (DQ156153 and DQ156154) by Reddy et al. (2007). The restriction enzymes predicted from sequences (HQ236382-HQ236388) obtained in the present study yielded expected RFLP pattern (Table 1) in all the samples that were analysed. Thus, it was concluded that PCR-RFLP using *Bam*HI or *Rsa*I restriction enzymes can be successful for the sex identification of the four vulture species of interest in our study. The PCR-RFLP method has previously been used for sex identification of the Short-toed Eagle (*Circaetus gallicus*) using *Hae*III for *CHD-Z* and *Asp*700I for *CHD-W* (Sacchi et al. 2004).

Another approach using universal gender identification *CHD-ZW* common and *W*-specific primers in combination with Griffith’s universal *CHD* primer P2 in two independent reactions has been reported earlier for Crested Serpent Eagles, where standard agarose gels were shown to easily distinguish between the 148 bp *CHD-ZW* and the 258 bp *CHD-W* PCR products (Chang et al. 2008b). These reported primers were aligned on vulture sequences obtained in the present study and were found suitable for molecular discrimination of sexes for *G. indicus* (n = 14), *G. bengalensis (*n = 28) and *G. himalayensis* (n = 3). This test was found suitable for several reasons- 1) easy interpretation of results in agarose gel as presence or absence of the *CHD W*-specific PCR product; 2) The PCR product size difference of 110 bp in *CHD-Z* and *CHD* W amplicons with these primers was far easier to differentiate in agarose gel than Griffith’s universal *CHD* primer pair P2/P8 (only 3–6 bp difference); 3) it can be employed for high throughput sex identification of vultures using real-time PCR combined with melting curve analysis, and 4) The PCR product obtained is relatively small size (153 from *CHD-Z* and 263 bp from *CHD-W*) and thus, permits the application of this test with degraded DNA samples. In our study, we could not get adequate results with Griffith’s universal *CHD* primer pair P2/P8 due to the large size of the expected product (approximately 390 bp) in some samples of poor quality genomic DNA, but the *W*-specific approach could prove useful in identifying sex of post-mortem samples. This *W*-specific PCR has also been shown to be an efficient and reliable method to identify sex of American Coots (*Fulica americana*) where *CHD-Z* polymorphism does not permit accurate sexing by traditional methods (Shizuka and Lyon 2008).

Further, sex determination using ARMS-PCR based on multiplexing of three primers namely, NP, MP and Griffith’s universal *CHD* primer P2, was evaluated. The primers NP and P2 yielded a single PCR product with male birds. Due to point mutations in *CHD-Z* and *CHD-W* sequences, one primer (MP) having 3’- mismatch with *CHD-Z* allele amplified product (293 bp) only with *CHD-W* allele and yielded two PCR products with female birds. This ARMS-PCR approach has been successfully reported for sex identification in a range of Falconiformes species (Ito et al. 2003; Chang et al. 2008a) and use of this approach in *G. bengalensis* and *G. indicus* has been indicated earlier but with some reservations (Reddy et al. 2007). However, in our study we have not only strengthened the applicability of this approach on *G. indicus* and *G. bengalensis* through validating the results on 26 birds of known sex, but also confirmed that the test can be used for *G. himalayensis* (female) and *A. monachus* (male). In our study, the test was found appropriate for male and female sex identification in dead as well as for live bird samples. However, the presence of only one nucleotide mismatch in primer (MP) for *CHD-Z* and *CHD-W* sometimes generated a faint *CHD-W*-specific band in males that may lead to some ambiguity with this method.

In real-time PCR using *CHD-W*-specific and *CHD-ZW*-common TaqMan probes, fluorescence for both probes was detected with female birds while, fluorescence for *CHD-ZW*-common probe was detected with males. In this study, real-time PCR using these probes was evaluated with tissues from dead birds and blood samples from live birds. This method is quick and robust for unambiguous sex determination in birds and has been utilised for gender identification of a large numbers of raptors (Chang et al. 2008c; Chou et al. 2010). The homology of these probes with *CHD-Z* and *CHD-W* sequences of *G. himalayensis* and *CHD-Z* sequence of *A. monachus* is reported in the present study and further application in a larger number of samples from these two species is warranted to validate this test.

All the molecular methods utilised in our study were employed to identify sex of 29 dead vulture samples. The sex identified by molecular methods for 17 tissue samples from dead vultures was found consistent with that known to the breeding centre for 13 samples. Repeated testing on four mismatched samples, reported in this study, produced the same results under a variety of different molecular sexing methods and we conclude that the original field post-mortems were incorrect in assigning sex. It appears that predicting sex at the time of necropsy may be error prone as gonads are reported to completely regress in birds during the non-breeding season (Hau 2001; Sharah et al. 2007; Nazrul Islam et al. 2012) and this situation may be aggravated when the carcass is putrefied. This might be the reason for the discrepancy in identification of sex from four dead vulture samples. All methods of sex identification for five live *G. indicus* and *G. bengalensis* birds and by the *CHD-W* specific method for an additional four birds were found to match with behavioural records of centre.

In conclusion, the molecular methods utilised in our study can be used to overcome some of the inherent problems of sexing during necropsy and used for accurate identification of sex in ecological studies. Further, these methods are useful for identifying the gender of live birds in the vulture conservation breeding centres and will thereby allow managers to keep balanced sex ratio in breeding aviaries. Among the methods, *S. c. hoya CHD-W* specific with *CHD-ZW* internal control primers in combination with Griffith’s universal P2 primer provided a relatively simple and robust test for sex identification in the three species of *Gyps* vultures for individual assays or for high-throughput sex identification and its utility is now being applied and used at the breeding centres in India (CZA, 2011).

## Materials and methods

### Sample collection and DNA isolation

A total of 46 individuals birds were used in the study. Permission to collect tissue samples from dead birds and blood samples from live birds (during routine health checks) at Vulture Conservation Breeding Centre, Pinjore, Haryana was approved by the State Forest Department, Haryana and Ministry of Environment and Forests, Government of India. Tissue samples were available from the necropsies carried out on 29 vulture carcasses. The sex of 17 birds (12 *G. bengalensis*, four *G. indicus* and one *A. monachus*) were identified during post mortem, however in the remaining 12 birds (six *G. bengalensis*, three *G. indicus* and three *G. himalayensis*) sex could not be identified. Blood samples were available from 17 birds from the BNHS Vulture Conservation Breeding Centre (VCBC), Pinjore, Haryana, of which 9 were of known-sex birds (six *G. bengalensis* and three *G. indicus*) and eight of unknown-sex (four *G. bengalensis* and four *G. indicus*). The sex of live birds in the breeding centres was identified based on their behaviour during copulation and egg laying. Tissue samples from dead birds were collected by trained veterinarians and the sex of all birds was identified during necropsies by visual identification of testes and ovaries.

Genomic DNA was isolated from various tissue types including: pectoral muscle, testes, ovary, crop, and gizzard collected during postmortem using QIAamp DNA mini kit (Qiagen, Valencia, CA, USA) and from blood samples collected over EDTA as an anticoagulant by QIAmp DNA Blood mini kit (Qiagen, Valencia, CA, USA) as per the manufacturers’ instructions. The quality of DNA was checked in 0.8% agarose gel electrophoresis.

### Sequence characterization for *CHD-Z* and *CHD-W* sequences

The Griffiths universal *CHD* primer pair P2/P8 (Griffiths 2000) was used to amplify the partial *CHD* gene from genomic DNA isolated from known-sex *G. bengalensis* female (P10) and male (P33); *G. indicus* female (P17) and male (P35); *A. monachus* male (P30) and *G. himalayensis* (P49) (unknown at the time of collection but identified as female in *W*-specific PCR). PCR reaction was performed in 25 μl reaction volume consisting of 0.4 μM each of P2 (Forward 5'-TCTGCATCGCTAAATCCTTT-3') and P8 (Reverse 5'-CTCCCAAGGATGAGRAAYTG-3') primers, 100–200 ng genomic DNA, 0.2 mM each dNTPs in 1x reaction buffer containing 2 mM MgCl_2_ and 1U Pfu UltraII Fusion HS DNA polymerase (Stratagene). No template control (NTC) containing no DNA was run with every PCR and precautions were taken to avoid cross-contamination. PCR cycle condition consisted of an initial denaturation at 94°C for 4 min, followed by 5 repeated cycles of 94°C for 30 sec, 49°C for 30 sec, 72°C for 30 sec; 49 repeated cycles of 94°C for 30 sec, 48°C for 20 sec, 72°C for 20 sec and final extension at 72°C for 5 min. The PCR products were separated on 3% agarose gel, purified using QIAquick gel extraction kit (Qiagen, Valencia, CA, USA) and cloned using CloneJET™ PCR Cloning Kit (Fermentas) following the manufacturer’s instructions. The recombinant plasmids were characterized and nucleotide sequences were determined using a T7 promoter primer. The nucleotide sequences for *CHD-Z* and *CHD-W* alleles from all species were aligned using MegAlign Lasergene software (DNAstar Inc, USA). Restriction endonuclease *Rsa*I and *Bam*HI sites were selected for sex identification in PCR-RFLP analysis.

### Standardization of PCR-based molecular methods for sex identification

i) Conventional PCR-RFLP

Using Griffiths universal *CHD* primer pair P2/P8, the amplified PCR products were analysed using restriction endonuclease digestion with *Rsa*I and *Bam*HI and sex was identified. The restriction digestion was performed in a 30 μl reaction volume containing 5 μl of amplified PCR product and 2 U of restriction enzymes (*Rsa*I or *Bam*HI) and was incubated at 37°C overnight. The digested products were separated on 3% agarose gel along with 100 bp DNA ladder and analysed.

ii) ARMS-PCR

ARMS-PCR based on 3’-terminal mismatch primer (MP primer) point mutation conserved among Falconiformes *CHD-W* and *CHD-Z* sequences previously reported (Ito et al. 2003) was performed to identify sex in vultures with some modifications. Briefly, PCR was done in a 25 μl reaction volume containing 0.4 μM each of Griffiths universal *CHD* primer P2 forward primer, another forward primer MP (5’-AGTCACTATCAGATCCGGAA-3’) and reverse primer NP (5’-GAGAAACTGTGCAAAACAG-3’), 100 ng genomic DNA, 0.2 mM each dNTP and 1U of Taq DNA Polymerase (Bangalore Genei, India). PCR amplification cycle involved initial denaturation at 94°C for 90 sec followed by 35 cycles of 94°C for 30 sec, 50°C for 45 sec, 72°C for 30 sec and final extention at 72°C for 5 min. The amplified PCR products were separated on 3% agarose gel along with 100 bp DNA ladder and analysed.

iii) *W*-specific PCR

An alternative *W*-specific sex identification method suggested for Crested Serpent Eagle (*Spilornis cheela hoya*) (Chang et al. 2008b) was also used in this study, where Griffith’s universal *CHD* primer P2 was used as a forward primer and *CHD-W* primer as a reverse primer which anneals to only the *CHD-W* allele sequence, or *ZW*-common primer which anneals to both *CHD-Z* and *CHD-W* allele sequences. The PCR reaction was performed in a 25 μl volume consisting of 0.4 μM each of Griffith’s universal *CHD* primer P2 and reverse primer *CHD-ZW*-common (5’-GATCAGCTTTAATGGAAGTGAAG-3’) or *CHD-W* specific (5’-GGTTTTCACACATGGCACA-3’), 100 ng genomic DNA, 0.2 mM each dNTP, 1.5 μl DMSO and 1U Taq DNA Polymerase (Bangalore Genei, India). The PCR cycling condition employed was an initial denaturation at 94°C for 3 min, followed by 45 repeated cycles of 94°C for 30 sec, 56°C for 30 sec, 72°C for 20 sec, and final extension at 72°C for 5 min. The amplified PCR products were resolved on 3% agarose gel along with 100 bp DNA ladder and analysed for presence (indicating female) or absence (indicating male) of 263 bp *W*-specific product.

iv) TaqMan probe based real-time PCR

The TaqMan based qualitative real-time PCR (qPCR) based on allele discrimination option for sex identification reported earlier for *S. cheela hoya* (Chang et al. 2008c) was used. This test utilises the considerable difference in composition of the *CHD-W* and *CHD-Z* sequences in vultures, with the *W*-specific probe (5’-FAM-TGTGCCATGTGTGAAAACCACCCA-TAMRA) recognising only the *CHD-W* region whereas the *ZW* common probe (5’-HEX-CCCTTCACTTCCATTAAAGCTGATCTGG-TAMRA) recognises both the Z and W *CHD* chromosome regions. The PCR reaction mixture in a 20 μl volume consisted of 0.4 μM each of Griffith’s universal *CHD* primer pair P2/P8, 50–100 ng genomic DNA, 0.2 mM of each dNTP, 20nM each of *W*-specific and *ZW* common probes and 1 U of Taq DNA polymerase (Bangalore Genei, India). The DNA template was excluded from no template control (NTC), whereas the probe was excluded from no probe control (NPC). In addition, positive controls (with known male and female DNA samples) were also included in each test. Two steps PCR condition was employed with initial denaturation at 94°C for 4 min, followed by 50 repeated cycles of 92°C for 15 sec, 60°C for 1 min in Mx3005P real-time PCR machine (Agilent, USA). The results were recorded as an amplification plot, with text report and alleles discrimination made using MxPro™ QPCR software (Agilent, USA) and compared with female and male positive controls.

### Application of the molecular methods for sex identification

All molecular methods were employed for sex identification of vultures using tissue samples obtained during necropsy (*n* = 17) and blood samples obtained from live birds (*n* = 9) for which the sex was known. These tests were then employed for analysing eight blood samples and 12 necropsy tissues from unknown-sex vultures.

## Abbreviations

*CHD*: Chromohelicase-DNA binding gene
IUCN: International Union for Conservation of Nature
NSAIDs: Non-steroidal antiinflammatory drugs
RFLP: Restriction Fragment Length Polymorphism
ARMS: Amplification Refractory Mutation System
SSCP: Single strand conformation polymorphism
*qPCR*: Qualitative real-time PCR
VCBC: Vulture Conservation Breeding Centre.
